# A Rare Gastric Subepithelial Lesion Removed through Submucosal Tunneling Endoscopic Resection: Case Report and Literature Review

**DOI:** 10.3390/life13010179

**Published:** 2023-01-08

**Authors:** Mu-Ming Chien, Yun-Ho Lin, Chun-Chao Chang, Hsi-Yuan Chien

**Affiliations:** 1 Department of Pediatrics, Taipei Medical University Hospital, Taipei 110, Taiwan; 2 TMU Research Center for Digestive Medicine, Taipei Medical University, Taipei 110, Taiwan; 3 Department of Pathology, Taipei Medical University Hospital, Taipei 110, Taiwan; 4 Division of Gastroenterology and Hepatology, Department of Internal Medicine, Taipei Medical University Hospital, Taipei 110, Taiwan

**Keywords:** glomus tumor, gastric subepithelial lesion, submucosal tunneling endoscopic resection

## Abstract

Gastric subepithelial lesions are common. However, their diagnosis and management can pose a challenge. Herein, we present the case of a 49-year-old man who was incidentally discovered to have a gastric subepithelial lesion that increased in size during follow-up. Submucosal tunneling endoscopic resection was performed, and the tumor was successfully removed en bloc. The pathological and immunohistochemical findings were consistent with a gastric globus tumor. Although rare, glomus tumors should be considered when gastric subepithelial lesions are discovered. Resection with an endoscopic technique can be used to preserve the stomach and can be considered an alternative to surgical removal. However, such procedures should only be performed by experienced therapeutic endoscopists.

## 1. Introduction

Gastric subepithelial lesions (SELs) are fairly common and have been reported to have a prevalence of 0.76–1.7% among the general population [1,2]. The initial management of gastric SELs hinges on proper diagnosis to determine whether the lesion has any malignant potential. A previous study reported that approximately 15% of such SELs are malignant [3].

According to the American Society of Gastrointestinal Endoscopy and European Society of Gastrointestinal Endoscopy guidelines, endoscopic ultrasound (EUS) is the most accurate imaging test for evaluating SELs and should be used to evaluate such lesions [4,5]. However, in many situations, EUS alone cannot distinguish all types of subepithelial tumors. When SELs are suggestive of gastrointestinal stromal tumor (GIST), with size >20 mm, or have high-risk stigmata, tissue diagnosis should be provided. For asymptomatic gastric SEL without a definite diagnosis, surveillance and diagnostic resection are both acceptable options [4,5].

Herein, we present a case of asymptomatic SEL that increased in size over the follow-up. After a discussion with the patient, the lesion was successfully removed through submucosal tunneling endoscopic resection (STER). The pathologic report revealed the lesion to be a glomus tumor.

## 2. Case Presentation

**Figure 1:**
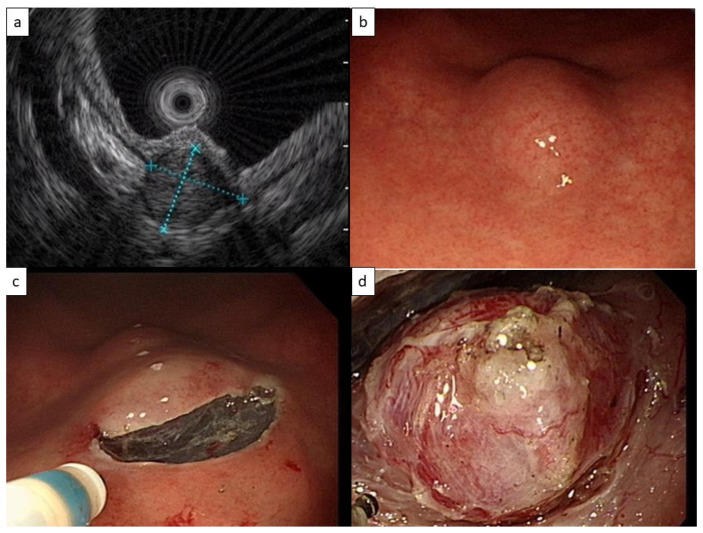
(**a**) Endoscopic ultrasound (11.0 × 11.8 mm^2^) of the heterogenous isoechoic tumor originating from the muscularis propria layer. (**b**) Esophagogastroduodenoscopy revealed a 20 mm submucosal tumor in the gastric antrum with apparently normal overlying mucosa. (**c**) An Olympus Dual-J endoscopic knife was used to cut through the mucosal and submucosal layers a few centimeters in front of the tumor to create a tunnel to the tumor. (**d**) The tumor was removed from the muscularis layer en bloc.

During a health examination, esophagogastroduodenoscopy revealed that a 49-year-old man had a 1.5 cm gastric submucosal tumor. EUS revealed a heterogenous isoechoic tumor with a size of 11.0 × 11.8 mm^2^ originating from the fourth echo layer, or the muscularis propria layer (Figure 1a). A GIST or leiomyoma was initially considered. No specific symptoms developed. The tumor increased to 2 cm in diameter over a 3-year period (Figure 1b), and STER was arranged.

A mucosal incision was made proximal to the tumor with an endoscopic knife (Dual-J knife, Olympus Tokyo, Japan) after submucosal injection of a glycerol solution. A tunnel was created using the submucosal dissection technique (Figure 1c). The tumor was then carefully dissected from the muscularis layer and removed en bloc (Figure 1d). The tunnel opening was closed with hemoclips. The patient tolerated the procedure well and was discharged from the hospital 2 days later.

**Figure 2:**
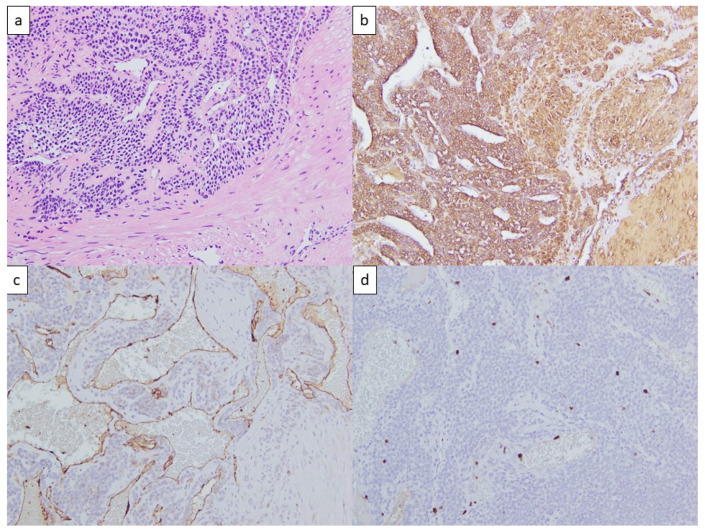
(**a**) Hematoxylin and eosin stains of the tumor revealing branching capillary-sized vessels lined by endothelial cells surrounded by collars of uniform tumor cells forming nests, sheets, and trabeculae; round neoplastic cell with indistinct borders and rounded nuclei. (**b**) Positive smooth muscle actin stain. (**c**) Negative CD31 stain. (**d**) Mitotic index of Ki67 less than 3%.

Histological study further revealed that the tumor comprised branching capillary vessels surrounded by collars of uniform tumor cells (Figure 2a). The round neoplastic cells had indistinct borders and a rounded nucleus in an amphophilic to eosinophilic cytoplasm. The neoplastic cells were positive for smooth muscle actin (Figure 2b) and negative for cytokeratin, S-100, CD31 (Figure 2c), CD34, chromogranin-A, and synaptophysin. Very rare mitoses were noted, and the mitotic index of Ki67 was less than 3% (Figure 2d). The final diagnosis was a benign glomus tumor.

## 3. Discussion

A glomus tumor is a mesenchymal neoplasm that can be found anywhere throughout the body. Such tumors are rarely reported in the stomach [6]. When a possible glomus tumor is discovered during endoscopic examination, two challenges with respect to management arise.

The first involves accurately diagnosing the tumor. Glomus tumors usually originate from the third or fourth echo layer and are mostly discovered in the antrum. Such tumors can appear hypoechoic or hyperechoic on EUS, with hypervascularity and internal echo patterns indicating calcification [4,7]. However, many of the characteristics of such tumors are similar to those of GISTs, leiomyoma, or schwannoma, and some glomus tumors may have atypical EUS features.

The management of SELs depends on precise diagnosis. Despite being the current best tool to characterize SEL, EUS alone is not able to distinguish among all the different types of SEL. For SELs with an unknown diagnosis, the most concerning diagnosis is GIST. The management of SELs with an unknown diagnosis in the different society guidelines generally reflects its policy regarding the management of GIST.

In Table 1, we listed the recommendations from the main societies in the field regarding the timing for tissue acquisition, management of small gastric GIST without high-risk features, and the management of gastric SELs with an unclear diagnosis. Most of the guidelines suggest tissue acquisition when GIST is suspected, or when size >20 mm as the malignant potential becomes higher. Nevertheless, obtaining a definite tissue diagnosis in some case is difficult, especially in lesions with a small size. In such cases, active surveillance is recommended, while diagnostic resection is a feasible option to prevent poor compliance of surveillance, and to decrease the burden of periodic endoscopy [4,7].

In our case, the gastric SEL was <20mm on initial presentation, originating from the muscularis propria layer but without high-risk features. Tissue acquisition or active surveillance were both feasible options. As the tumor size gradually increased over time, tissue acquisition to confirm the diagnosis or diagnostic resection were both acceptable management techniques. As there are still many controversies regarding the management of small gastric SELs, the decision depends on the availability of local resources, the endoscopist’s experience, and the patient’s preference.

Even if tissue acquisition was performed in our case, the correct diagnosis might not have been made. In a recent review, 46 cases of gastric glomus tumor were reported in the literature between 2010 and 2019, and only 19 of them had a preoperative diagnosis. Among the patients who underwent endoscopic ultrasound-guided fine-needle aspiration to determine the pathology, a diagnosis was correctly given in only 8 out of 13 cases [13]. Although EUS-guided biopsy has been reported to be able to provide an accurate diagnosis in some cases, resection might be a more favorable alternative for both diagnosis and treatment [4,14].

The second challenge related to management involves the uncertainty of the pathological nature of glomus tumors. Glomus tumors are typically benign, and the guidelines released by the European Society of Gastrointestinal Endoscopy in 2022 indicate that no surveillance is required if the diagnosis is clear because only anecdotal evidence supports that a risk of malignancy or complications is present [4]. However, in a systematic review of 187 cases of gastric glomus tumors, 11 cases involved malignant glomus tumors [14].

Criteria were proposed for defining malignancy in glomus tumors originating from soft tissues [15]. In addition, for gastroesophageal glomus tumors, researchers have proposed that tumors ≥5 cm or with both atypia and mitoses ≥2/10 HPF should be considered malignant and that copy number analysis might be helpful in borderline cases [16].

Because glomus tumors are potentially malignant, if removal is planned, a local operation for complete resection is typically recommended, and such operations are indeed performed in most cases in the literature [14]. However, surgical resection often requires sacrificing a considerable amount of stomach tissue. Some studies have reported on the removal of glomus tumors through endoscopic enucleation or endoscopic submucosal dissection (ESD) [17,18,19]. STER is an emerging method for removing submucosal lesions. STER enables wide visualization of the submucosal layer, which more effectively exposes the submucosal lesion. In a study comparing STER and conventional ESD to treat early gastric cancer, STER was reported to involve faster resection and a lower incidence of perforation [20]. In cases of SELs, a potential advantage of STER over ESD is its ability to maintain mucosal integrity. Because the mucosa covers the defect after the removal of the lesion, wound healing can be promoted, and the chance of delayed perforation is potentially lessened [5]. In addition, when perforation occurs, the defect can be covered by the mucosa to prevent further leakage.

Regardless of which endoscopic technique is considered, procedures should always be performed by an endoscopist skilled in advanced tissue resection techniques, as suggested by the American Gastroenterological Association [8].

## 4. Conclusions

Gastric globus tumors are a rare form of gastric subepithelial lesion. With the current case, we demonstrated the possible challenges in the clinical scenario, including the difficulties in correctly diagnosing such lesions, the timing of tissue acquisition, and the uncertainty of the pathological nature. If the removal of gastric globus tumors is planned, previously it was mainly performed by local operation, with some reports using ESD. We showed that STER may have some potential advantages over ESD and can be an alternative. Compared to an operation, resection with an endoscopic technique can better preserve the stomach, but should always be performed by an experienced endoscopist.

## Figures and Tables

**Table 1:** Recommendations from different society guidelines regarding timing for tissue acquisition, management of small GIST (<20 mm) and SELs with unknown diagnosis.

Society, Year	Timing for Tissue Acquisition	Gastric GIST, <20 mm, without High-Risk Features 1	Gastric SELs with Unclear Diagnosis
ESGE, 2022 [4]	All SELs with features suggestive of GIST Size > 20 mm Have high-risk stigmata When surgical resection or oncological treatment is required	Surveillance or resection	<10 mm EGD at 3–6 months, then at 2–3 years interval 10–20 mm EGD at 3–6 months, then at 1–2 years interval. Diagnostic resection is an alternative for SELs <20 mm after failure of attempts to obtain diagnosis >20 mm EGD + EUS at 6 months, then at 6–12 months interval
AGA, 2022 [8]	Lesion arising from muscularis propria layer	Surveillance with EUS, 1 year interval	Not specifically mentioned
ESMO–EURACAN–GENTURIS, 2022 [9]	Size > 20 mm	Resection. Surveillance is an alternative	<20 mm Active surveillance. short interval (e.g., 3 months) then increased interval. Resection as an alternative ≥ 20 mm Biopsy/excision
NCCN, 2022 [10]	When surgical resection or oncological treatment is required	Periodic endoscopic or radiographic surveillance. Risk and benefit should be discussed with the patient	Not specifically mentioned
ASGE, 2017 [5]	Lesions arising from submucosal or muscularis propria layer	Surveillance with EUS, 6–12 months interval	Removal as an alternative to tissue acquisition
Asian consensus guidelines for GIST, 2016 [11]	When surgical resection or oncological treatment is required	Resection. Surveillance is an alternative after informing the risk of malignancy	Not specifically mentioned
Japan GIST guideline subcommittee, 2008 [12]	Not specifically mentioned	Resection	<20 mm EGD at 6–12 months interval. When tumor growth or high-risk feature is noted, further examination is suggested, while resection is an alternative 20–50 mm Meticulous examinations with CT, EUS, and EUS-FNAB

AGA: American Gastroenterology Association; ASGE: American Society of Gastrointestinal Endoscopy; EGD: esophagogastroduodenoscopy; ESGE: European Society of Gastrointestinal Endoscopy; ESMO–EURACAN–GENTURIS: The European Society for Medical Oncology–European Reference Network for Rare Adult Solid Cancers–European Reference Network for Genetic Tumour Risk Syndromes; EUS: endoscopic ultrasound; EUS-FNAB: endoscopic ultrasonography-guided fine-needle aspiration biopsy; ^1^ high-risk features includes: irregular border, cystic spaces, ulceration, echogenic foci, and heterogeneity.

## Data Availability

The data are not publicly available due to ethical restriction.
